# Using dynamic point light display stimuli to assess gesture deficits in schizophrenia

**DOI:** 10.1016/j.scog.2022.100240

**Published:** 2022-02-03

**Authors:** Anastasia Pavlidou, Victoria Chapellier, Lydia Maderthaner, Sofie von Känel, Sebastian Walther

**Affiliations:** University of Bern, University Hospital of Psychiatry and Psychotherapy, Translation Research Center, Bern, Switzerland

**Keywords:** PLD, Psychosis, Communicative gestures, Social communication, Dynamic stimuli

## Abstract

**Background:**

Gesture deficits are ubiquitous in schizophrenia patients contributing to poor social communication and functional outcome. Given the dynamic nature of social communications, the current study aimed to explore the underlying socio-cognitive processes associated with point-light-displays (PLDs) of communicative gestures in the absence of any other confounding visual characteristics, and compare them to other well-established stimuli of gestures such as pictures by examining their association with symptom severity and motor-cognitive modalities.

**Methods:**

We included 39-stable schizophrenia outpatients and 27-age-gender matched controls and assessed gesture processing using two tasks. The first task used static stimuli of pictures of a person performing a gesture. The limbs executing the gesture were missing and participants' task was to choose the correct gesture from three-options provided. The second task included videos of dynamic PLDs interacting with each other. One PLD performed communicative gestures, while the other PLD imitated/followed these performed gestures. Participants had to indicate, which of the two PLDs was imitating/following the other. Additionally, we evaluated symptom severity, as well as, motor and cognitive parameters.

**Results:**

Patients underperformed in both gesture tasks compared to controls. Task performance for static stimuli was associated with blunted affect, motor coordination and sequencing domains, while PLD performance was associated with expressive gestures and sensory integration processes.

**Discussion:**

Gesture representations of static and dynamic stimuli are associated with distinct processes contributing to poor social communication in schizophrenia, requiring novel therapeutic interventions. Such stimuli can easily be applied remotely for screening socio-cognitive deficits in schizophrenia.

## 1 Introduction

Schizophrenia is a severe psychiatric disorder characterized by hallucinations, delusions, conceptual disorganization, impaired motor abnormalities, and negative symptoms. Social withdrawal and affective flattening are negative symptoms, which impair cognitive tasks and social communication leading to severe deficits in social functioning and poor quality of life (Green et al., 2015; McCutcheon et al., 2020; Walther et al., 2015; Wible, 2012b). Social communication is largely dependent on social interaction processes and its success strongly relies in correctly interpreting, perceiving and responding to socially relevant stimuli (Green et al., 2008). Schizophrenia patients exhibit severe deficits in correctly identifying social cues (i.e., agency, facial expressions, gestures and intention (Green et al., 2015; Wible, 2012b)). Gestures, the main focus of this study, are particularly affected (Walther and Mittal, 2016). Gestures are visible bodily-movements used alone or in conjunction with speech to aide social communication (Goldin-Meadow, 1999). Thus, successful social communication strongly relies in accurately performing and interpreting gestures.

Performance and perception of gestures in schizophrenia are highly correlated. For example, schizophrenia patients with impaired gesture performance also exhibit deficits in tasks involving nonverbal social perception (i.e., facial expression), gestural knowledge and tool-use suggesting a generalized impairment in nonverbal communication related to gestures in this population (Walther et al., 2015). Errors in gesture performance during imitation involve both spatial-temporal configurations, including extra movements, omissions, spatial orientation errors, and body-part-as-object errors (Walther et al., 2020c; Walther et al., 2013a). In addition, schizophrenia patients use fewer gestures during social communications (Lavelle et al., 2015; Lavelle et al., 2013) while clinical-high-risk subjects tend to use gestures in the wrong context (Millman et al., 2014). Similarly, schizophrenia patients show deficits in interpreting and recognizing hand gestures (Bucci et al., 2008; Walther et al., 2015; White et al., 2016), as well as, in the ability to discriminate between types of gestures that are congruent or incongruent with speech (Choudhury et al., 2021; Nagels et al., 2019). These gestural deficits are linked to structural and functional abnormalities within the praxis network of the brain (Choudhury et al., 2021; Stegmayer et al., 2016; Stegmayer et al., 2018; Viher et al., 2018; Wuthrich et al., 2020), which includes frontal-parietal-temporal cortices (Yang et al., 2015). Correct gesture processing is strongly dependent on the occurring interactions between these areas (Viher et al., 2020).

Gesture abnormalities in schizophrenia are also associated with symptom severity. For example, impairments in gesture performance predict poor functional outcome and capacity after six months and are related to both positive and negative symptoms, as well as, motor deficits (Dutschke et al., 2018; Lavelle et al., 2013; Matthews et al., 2013; Nagels et al., 2019; Park et al., 2008; Walther et al., 2015; Walther et al., 2013a, Walther et al., 2013b). In addition, deficits in gesture perception are linked to positive symptoms (Walther et al., 2015) and formal thought disorder (Nagels et al., 2019; Walther et al., 2020a). Furthermore, gesture deficits are associated with working memory abilities and frontal lobe dysfunction (Walther et al., 2015; Walther et al., 2013b).

To date, studies examining gestures in schizophrenia heavily relied on stimuli such as, pictures of hand gestures (static stimuli) or video-recordings of real-life-agents acting out different gestures (Straube et al., 2011; Walther et al., 2015). Given the dynamic nature of social interactions, static stimuli offer minimal ecological validity, while video-recordings require the process of multiple verbal and nonverbal-cues (posture and gaze direction) that reflect several underlying socio-cognitive processes that go beyond gesture perception (Okruszek, 2018). In addition, likability and gender of the agent may affect the perception of these stimuli (Mehta et al., 2011). These limitations may influence how we interpret the link between gesture deficits and psychopathology. One way to limit these confounding effects is to use Point-light-displays (PLDs; (Okruszek, 2018)). PLDs are dynamic stimuli that depict biological movements in the absence of any visual properties. These movements are created by placing point-light dots on the main joints of a human actor (Johansson, 1973). Viewers are able to perceive motion solely from the movement of the dots, successfully distinguishing between natural and unnatural movements (Pavlidou et al., 2014), emotional states (Alaerts et al., 2011), male and female actors (Mather and Murdoch, 1994), as well as, communicative and non-communicative movements (Manera et al., 2011a; Manera et al., 2011b).

Schizophrenia patients exhibit deficits in discriminating biologically relevant information provided by PLDs (Jahshan et al., 2015; Kern et al., 2013; Kim et al., 2005; Kim et al., 2013; Okruszek and Pilecka, 2017) and are worse in identifying the actions of two PLD agents (Okruszek et al., 2018). However, the association between these types of dynamic stimuli and symptom severity, as well as, motor and cognitive impairments in schizophrenia, and how these associations compare to other stimuli of gestures requires further investigation.

The current study aims to explore the underlying symptoms and processes associated with task performance during the perception of pictures of hand gestures (static stimuli), and during the interpretation of communicative gestures between two PLD agents (dynamic stimuli). We expect schizophrenia patients to underperform in both tasks compared to controls. In addition, we expect performance in these tasks to be associated with symptom severity, motor and cognitive impairments. However, due to the dynamic nature of the PLD stimuli and the fact that participants are not actively involved in the ongoing social interaction between the two PLD agents, participants adopt a third-person perspective. We expect these stimuli to be involved in processes that integrate multisensory cues important for understanding social relationships. Such processes depend not only on motor imagery and on motor simulation but also on perspective-taking and social salience (Frith and Frith, 2006; Wenke et al., 2011).

## 2 Materials and methods

### 2.1 Participants

We included 39 stable outpatients (56.4% males) and 27 controls (48.1% males) matched for age and gender that are part of a larger ongoing study (Brain Stimulation And Group Therapy to Improve Gesture and Social Skills in Psychosis trial, clinicaltrials.gov NCT04106427). All participants were right-handed in accordance to the Edinburgh Handedness Inventory (Oldfield, 1971), and were native German speakers who had grown up in Switzerland. Demographic and clinical characteristics for each group are given in Table 1. Written informed consent was attained from all participants and the study was approved by the local ethics committee (2019-00798), and thus complies with the tenets of the Declaration of Helsinki. Patients were recruited from the outpatient department at the University Hospital of Psychiatry and Psychotherapy in Bern Switzerland. All patients were diagnosed with schizophrenia spectrum disorders (87% schizophrenia and 13% schizoaffective disorder) according to the DSM-5 criteria using the Mini International Neuropsychiatric Interview. At the time of testing, 85% of the patients were on antipsychotic medication, while 44% were on antidepressant medication, 13% received medication for high blood pressure, 5% for cardiovascular issues and 5% for diabetes. Controls were recruited through leaflets, advertisements, and word-of-mouth. Inclusion criteria for all participants: 18–65 years of age, no substance abuse (excluding nicotine) and no history or current neurological disorders. Additionally, for controls, no history of psychotic disorders and no first-degree relatives diagnosed with schizophrenia spectrum disorders.

**Table 1:** Demographic and clinical characteristics.

	Patients (*n* = 39)	Controls (*n* = 27)	Chi-square
Demographic			
Age (years)	39.0 ± 2.31	39.8 ± 1.92	χ^2^ = 0.12; *p* = .73
Gender (males %)	56.43%	48.12%	χ^2^ = 0.20; *p* = .65
Education (years)	14.1 ± 0.64	16.1 ± 0.52	χ^2^ = 7.57; *p* < .01⁎
Medication (CPZ-eq mg)	507.7 ± 74.94		

Clinical			
PANSS positive^b^	11.8 ± 0.70		
PANSS negative^b^	16.8 ± 1.16		
PANSS total^b^	58.8 ± 2.81		
BNSS total^b^	27.5 ± 2.41		
TALD total^b^	17.4 ± 1.99		

Motor			
NES total^a^	12.6 ± 1.21	4.7 ± 0.81	χ^2^ = 22.41; *p* < .01⁎
Sensory integration	2.6 ± 0.40	1.1 ± 0.30	χ^2^ = 8.03; *p* < .01⁎
Motor coordination	2.6 ± 0.33	1.1 ± 0.93	χ^2^ = 8.02; *p* < .01⁎
Sequencing of motor acts	4.3 ± 0.64	1.7 ± 0.38	χ^2^ = 6.77; *p* < .05⁎
Others	3.2 ± 0.52	0.7 ± 0.22	χ^2^ = 7.69; *p* < .01⁎

Cognition			
DSB	4.3 ± 0.22	4.5 ± 0.21	χ^2^ = 0.93; *p* = .34
MSCEIT-emotional management	87.6 ± 1.44	93.0 ± 1.56	χ^2^ = 6.47; *p* < .05⁎
MSCEIT-social management	87.8 ± 1.53	95.0 ± 1.45	χ^2^ = 11.29; *p* < .01⁎
MSCEIT-managing emotions branch	86.5 ± 1.57	94.1 ± 1.53	χ^2^ = 11.14; *p* < .01⁎

Values represent the mean ± SEM for each group. BNSS: Brief Negative Symptom Scale; DSB: Digit Span Backwards; MSCEIT: Mayer-Salovey-Caruso Emotional Intelligence Test; NES: Neurological Evaluation Scale; PANSS: Positive And Negative Syndrome Scale; TALD: Thought And Language Disorder.

a 3 patients did not complete NES.

b Was not assessed in controls.

⁎ Denotes a significant difference between patient and control group.

### 2.2 Gesture domains

#### 2.2.1 Static stimuli

**Fig. 1:**
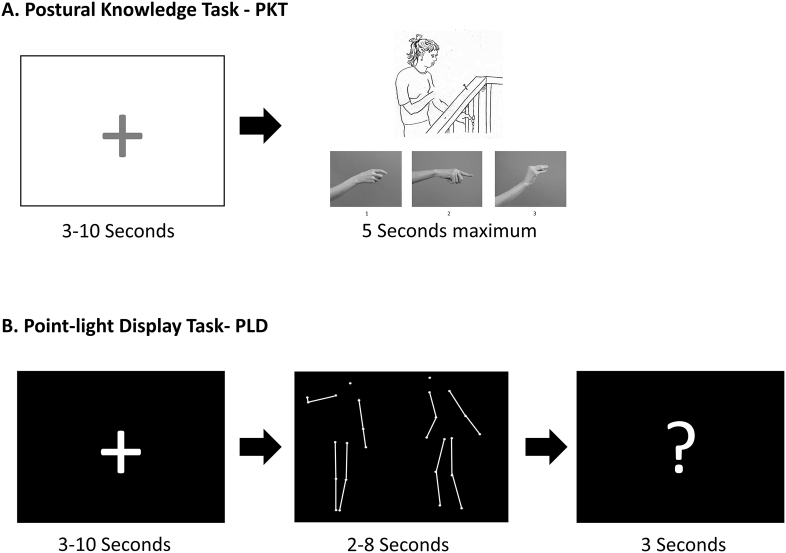
Experimental setup. (A) Postural knowledge task (PKT). Each trial started with a fixation cross (3–10 s) followed by a picture (5 s maximum) where the distal parts of the limbs performing a particular gesture are removed. Participants' task is to choose the correct gesture from the three options provided below using a button press (1, 2, and 3) and respond while the picture is still present. In the example provided the correct gesture is number one. (B) Point-light displays (PLD). Each trial started with a fixation cross (3–10 s) followed by a PLD animation, after which a question mark appeared (3 s). The PLD animation comprised of two PLDs one on the right of screen and the other on the left side. Throughout the course of the animation, one PLD was performing a communicative gesture while the other PLD would either imitate or follow these performed gestures. Participants' task is to indicate with a button press which of the two PLDs (left or right) is imitating or following the gestures of the other when the question mark is present. Connecting lines were not present in the actual experiment.

The Postural Knowledge Task (PKT; Fig. 1A) examines gestural knowledge (Mozaz et al., 2002). Performance of both patients and controls was measured using 20 pictures of people carrying out gestures while the distal parts of the limbs performing these gestures are missing. Below each picture, three possible limb positions are shown, and participants are asked to choose the correct one via a button press (1, 2 or 3). Each trial started with a fixation cross (3–10 s) followed by a picture (maximum 5 s). Participants were asked to respond when the picture was still present. Once participants responded a new trial begun. The starting picture was randomized across participants. The score for PKT ranges between 0 and 20, with higher values indicating superior performance.

#### 2.2.2 Dynamic stimuli

The point-light display (PLD) task (Fig. 1B) examined patients' and controls' ability to disentangle the occurring dynamic interactions between two PLD agents communicating with each other using different gestures (Manera et al., 2011a). Forty videos (available online) showed two PLD agents; one on the right side and the other on the left side of the screen. One PLD agent performed communicative gestures in either an informative (‘jumping’) or an instructive manner (‘please sit down’) while the other PLD agent was shown to imitate or follow these performed gestures. Participants had to indicate with a button press (left or right), which of the two PLDs imitated/followed the other. Each trial started with a fixation cross (3–10 s) after which a PLD animation appeared (2–8 s), followed by a question mark (3 s). Participants were asked to respond when the question mark appeared; once they did a new trial begun. The starting PLD animation was randomized across participants. The score for the PLD task ranges from 0 to 40, with higher scores indicating better performance.

### 2.3 Motor abnormalities

The Neurological Evaluation Scale (NES; (Buchanan and Heinrichs, 1989)) was used to assess neurological soft signs in both patients and controls. The scale includes 26-items focusing on sensory integration, motor coordination and sequencing of motor acts. A fourth category known as the ‘other’ includes eye-movement abnormalities, short-term memory and frontal-release signs. Higher scores indicate more impairment.

### 2.4 Cognition

We used the Digit Span Backwards (DSB; (Hilbert et al., 2015)) to assess working-memory abilities by testing patients' and controls' capacity to retain information in short-term memory. In addition, we used the Mayer-Salovey-Caruso Emotional Intelligence Test (MSCEIT; (Mayer et al., 2002)) to measure participants' ability to perceive and act on emotional information. Higher performance indicates better emotional perception.

### 2.5 Clinical assessments

To assess current symptom severity in patients we used the Positive And Negative Syndrome Scale (PANSS; (Kay et al., 1987)), the 13-item Brief Negative Symptom Scale organized into 6-subscales: anhedonia, distress, social withdrawal, avolition, blunted affect and alogia (BNSS; (Kirkpatrick et al., 2011)), and the Thought and Language Disorder (TALD) scale (Kircher et al., 2014).

### 2.6 Data analyses

Mean number of correct responses for both PKT and PLD tasks were calculated using scripts written in R (version 4.0.2); these values were not normally distributed, and thus we applied a log transformation. Demographic, clinical and cognitive data available for both patients and controls were compared using chi-square tests. Since education was significantly different between patients and controls (χ^2^ = 7.6; *p* < .01; Table 1) it was used as a covariate in our main analyses. In addition, performance of the PKT was further added as a covariate when comparing PLD performance between groups to control for deficits in gestural knowledge. We compared group performance for both tasks using parametric ANCOVA. We used Kendall correlations to explore patient-group associations between task performance and clinical, motor and cognitive parameters, while controlling for antipsychotic medication.

## 3 Results

### 3.1 Demographic and clinical data

Overall, patients and controls did not differ in age or gender but did in education with controls having more years of education than patients (Table 1). Patients had moderate symptom severity.

### 3.2 Gesture domains

**Fig. 2:**
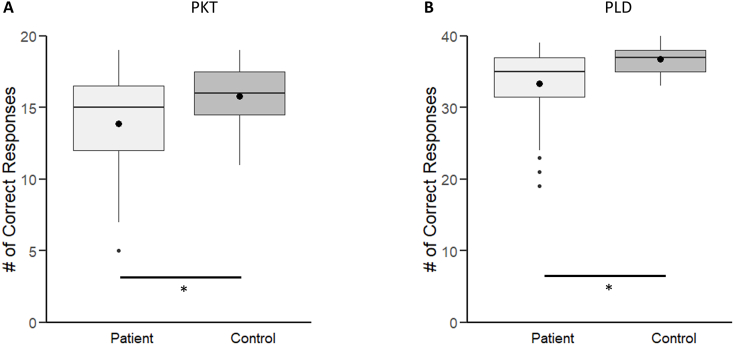
Results. Box plots comparing correct number of responses separately for the PKT (A) and PLD (B) tasks for both the patient (light grey) and the control (dark grey) groups. The thick horizontal line inside the box represents the median; while the black dot represents the mean. The upper and lower bound of each box represent the 75th and 25th percentiles of the distribution, while the top and bottom ends of the whisker represent the 95th and 5th percentiles of the distribution, respectively. * denotes a significant difference between patients and controls.

Patients performed worse than controls during the PKT (F _(1, 64)_ = 4.9, *p* < .05; Fig. 2A) task, even after controlling for education, pointing to severe deficits in gestural knowledge. Likewise, patients underperformed in the PLD task even after controlling for education and gestural knowledge (as measured by the PKT; F _(1, 63)_ = 4.4, *p* < .05; Fig. 2B). Furthermore, there was a strong significant association between task performance of PKT and PLD (tau = 0.32, *p* = .005).

### 3.3 Motor abnormalities

Patients had more neurological soft signs than controls (χ^2^ = 22.4, *p* < .001; Table 1). This was true across all categories: sensory integration, motor coordination, sequencing of motor acts and others (all χ^2^ > 6.5, all *p* < .05; Table 1).

### 3.4 Cognition

Patients and controls did not differ in DSB performance (χ^2^ = 0.9, *p* = .34; Table 1), however, emotional perception (MSCEIT) was significantly lower for patients (all χ^2^ > 6.6, all *p* < .05; Table 1).

### 3.5 Gesture domains and clinical parameters

**Fig. 3:**
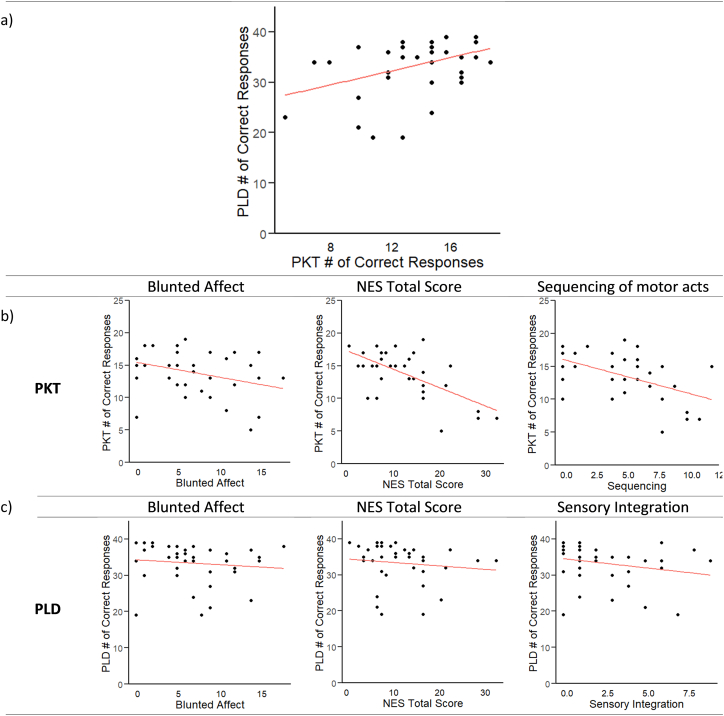
Schematic illustration of correlations in patients. (a) Positive correlation between PLD and PKT task performances (tau = 0.32, *p* < .01). (b) PKT task performance correlations with Blunted Affect (tau = −0.25, *p* < .05), NES total score (tau = −0.33, *p* < .01), and Sequencing of Motor Acts (tau = −0.37, *p* < .01). (c) PLD task performance correlations with Blunted Affect (tau = −0.21, *p* = .07), NES total score (tau = −0.22, *p* = .06), and Sensory Integration (tau = −0.25, *p* < .05).

Performance of both PKT and PLD tasks was not associated with symptom severity measured using PANSS, BNSS total and TALD (all tau < 1.6 all *p* > .13; Table 2). Exploring associations with specific subdomains of the BNSS revealed a significant negative correlation between PKT and blunted affect (tau = −0.3, *p* < .05; Table 3 and Fig. 3), while this correlation was at trend level for the PLD task (tau = −0.3, *p* = .07; Table 2 and Fig. 3). Further investigation into the specific items of the blunted affect domain with particular attention given to the expressive gesture item, revealed a significant negative correlation between PLD performance and expressive gestures (tau = −0.3, *p* < .05; Table 3). This correlation was absent for the PKT (tau = −0.18, *p* = .11; Table 3).

**Table 2:** Partial correlations for patients controlling for medication.

PKT		PLD	
Correlations	tau; *p*-value	Correlations	tau; p-value
Gesture domains and clinical parameters			
PKT-PANSS positive	0.09; 0.38	PLD-PANSS positive	0.17; 0.13
PKT-PANSS negative	−0.16; 0.15	PLD-PANSS negative	−0.00; 0.97
PKT-PANSS total	−0.04; 0.67	PLD-PANSS total	0.05; 0.60
PKT-BNSS total	−0.10; 0.36	PLD-BNSS total	−0.08; 0.44
PKT-TALD	0.03; 0.79	PLD-TALD	0.06; 0.58

Gesture domains and motor abnormalities			
PKT-NES	−0.33; <0.01⁎	PLD-NES	−0.22; 0.06

Gesture domains and cognition			
PKT-DSB	−0.03; 0.75	PLD-DSB	0.13; 0.23
PKT-MSCEIT emotional management	0.14; 0.19	PLD-MSCEIT emotional management	−0.01; 0.90
PKT-MSCEIT social management	0.11; 0.34	PLD-MSCEIT social management	0.02; 0.81
PKT-MSCEIT emotion branch	0.18; 0.12	PLD-MSCEIT emotion branch	0.03; 0.77

BNSS: Brief Negative Symptom Scale; DSB: Digit Span Backwards; MSCEIT: Mayer-Salovey-Caruso Emotional Intelligence Test; NES: Neurological Evaluation Scale; PANSS: Positive and Negative Syndrome Scale; PKT: Postural Knowledge Test; PLD: Point-light Displays; TALD: Thought and Language Disorder.

⁎ Denotes a significant correlation.

**Table 3:** Partial correlations for patients within BNSS and NES sub-domains controlling for medication.

PKT		PLD	
Correlations	tau; p-value	Correlations	tau; p-value
BNSS			
PKT-BNSS anhedonia	0.07; 0.49	PLD-BNSS anhedonia	−0.02; 0.84
PKT-BNSS distress	−0.10; 0.39	PLD-BNSS distress	−0.13; 0.23
PKT-BNSS social withdrawal	−0.18; 0.11	PLD-BNSS social withdrawal	0.03; 0.78
PKT-BNSS avolition	0.13; 0.24	PLD-BNSS avolition	−0.00; 0.98
PKT-BNSS blunted affect	−0.25; 0.02⁎	PLD-BNSS blunted affect	−0.21; 0.07
PKT-BNSS alogia	−0.08; 0.46	PLD-BNSS alogia	−0.04; 0.70
PKT-BNSS expressive gestures item	−0.18; 0.11	PLD-BNSS expressive gestures item	−0.25; 0.02⁎

NES			
PKT-NES sensory integration	−0.16; 0.16	PLD-NES sensory integration	−0.25; 0.02⁎
PKT-NES motor coordination	−0.27; 0.02⁎	PLD-NES motor coordination	−0.01; 0.95
PKT-NES sequencing of motor acts	−0.37; <0.01⁎	PLD-NES sequencing of motor acts	−0.17; 0.13
PKT-NES other	−0.15; 0.18	PLD-NES other	−0.18; 0.13

BNSS: Brief Negative Symptom Scale; NES: Neurological Evaluation Scale; PKT: Postural Knowledge Test; PLD: Point-light Displays.

⁎ Denotes a significant correlation.

### 3.6 Gesture domains and motor abnormalities

More neurological soft signs were associated with poorer PKT performance for patients (tau = −0.33, *p* < .01; Table 2 and Fig. 3). This association is strongly related to the motor coordination (tau = −0.27, *p* < .05; Table 3) and sequencing of motor acts (tau = −0.37, *p* < .01; Table 3 and Fig. 3) domains. A similar pattern was observed for PLD (tau = −0.22, *p* = .06; Table 2), however this association was strongly related to the sensory integration (tau = −0.25, *p* < .05; Table 3 and Fig. 3) domain.

### 3.7 Gesture domains and cognition

Performance of both PKT and PLD tasks was not associated with working-memory abilities (all tau < 0.13, all *p* > .23; Table 2) or emotional perception (all tau < 1.7, all *p* > .12; Table 2) as measured by the DSB and MSCEIT respectively.

## 4 Discussion

The current study aimed to explore gesture perception in schizophrenia, testing task performance of static and dynamic stimuli, and their association with symptom severity, motor and cognitive impairments. We report four main findings. First, as expected, patients underperformed during the perception and interpretation of static and dynamic stimuli of gesture representations compared to controls. Second, task performance in the PLD task was associated with specific BNSS items of the blunted affect subdomain, namely gesture expression. Third, poorer task performance in both tasks was associated with more neurological soft signs. The PLD task strongly correlated with the sensory integration subdomain of the NES whereas, the PKT task strongly correlated with the motor coordination and sequencing domains. Finally, gestural deficits in this study were not associated with other symptom domains as measured by the PANSS and TALD scales, or with cognitive processes such as working memory and emotion perception abilities.

Our first finding confirms previous reports that schizophrenia patients show deficits during the perception of gestural knowledge and during the interpretation of communicative gestures involving two agents interacting with each other compared to controls (Okruszek et al., 2018; Walther et al., 2015). Since different gesture representations depend on the integration of several multimodal cues related to symptoms, motor, sensory, social, and cognitive domains, deficits in gesture processing indicate dysfunction in one or more of these processes (Gupta et al., 2021). Thus, we explored the contributing factors that might be involved during the process of static and dynamic gesture representations.

Gesture deficits in both tasks are associated with a specific subdomain of the BNSS, namely blunted affect. Blunted affect involves expression of emotions, including facial, vocal and gesture expression, and is a major symptom in schizophrenia (Marder and Galderisi, 2017). A strong association between poor PLD performance and expressive gestures was observed in this study. Expressive gestures include the gestures made using the hands, shoulders, trunk and head of a human agent. During social communication, expressive gestures help in defining agency, social salience and intentions. Although, previous studies have reported a reduction of expressive gesture use in schizophrenia patients during interactions with psychiatrists (Brüne et al., 2008; Lavelle et al., 2013), our results extend this finding and suggest that this association is also present during the interpretation of others' gestures. In contrast, poor PKT performance was not tied to the expressive gesture item. Blunted affect in schizophrenia patients has been suggested to be linked to deficits in nonverbal social perception and/or motor abnormalities. In fact, evidence-showing associations between PKT performance and nonverbal social perception/motor deficits already exists (Walther et al., 2015). The association between these deficits is attributed to aberrant function of the mirror-neuron-mechanisms of gesture perception in schizophrenia patients and may underlie patients' difficulty in successfully imitating gestures (Lee et al., 2014).

In line with multiple reports, schizophrenia patients had more neurological soft signs than controls (Bachmann and Schröder, 2018) and this was strongly associated with poor performance in both tasks. This finding corroborates several studies that show strong associations between gesture deficits and motor abnormalities (Dutschke et al., 2018; Walther et al., 2015; Walther et al., 2013a). Interestingly, the current study revealed that each task was associated with specific subdomains of the NES scale. Poor PLD performance was associated with more deficits in the sensory integration domain. Sensory integration involves the combination of multiple sensory-cues and is known to take place in the temporo-parietal junction (TPJ) and posterior superior temporal sulcus (pSTS) of the brain (Decety and Lamm, 2007). Both TPJ/pSTS play a key role in the perception of dynamic stimuli involving social and expressive gestures of self-and-others. This involves one's ability to predict others' movements and interpret agency. Dysfunction in the sensory integration processes within TPJ/pSTS might explain the observed deficits in interpreting communicative gestures between two PLD agents in this study (Wible, 2012a). In contrast, poor PKT performance was associated with poor motor coordination and sequencing domains. Deficits in these domains are associated with aberrant activity of the mirror-neuron-system, and strongly related to correct motor planning and imitation of gestures (Walther et al., 2015).

Contrary to our expectation, gesture impairments in our study were not associated with severity of total positive and negative symptoms as previously reported. This might be related to the fact that our patients are more stable and exhibit less severe symptoms (lower PANSS scores) compared with the other studies (Walther et al., 2020b; Walther et al., 2015; Walther et al., 2013b). In addition, the current study did not observe any associations between gesture deficits and formal thought disorder (Nagels et al., 2019; Walther et al., 2020a). This might be due to the type of stimuli used, as previous studies show formal thought disorder is associated with the processing of abstract gestures. Further investigation is necessary. Furthermore, although schizophrenia patients often exhibit deficits in working-memory abilities (Walther et al., 2015), DSB performance of our patients was similar to controls, and thus was not associated with poor task performance. Finally, although patients exhibited inferior emotion perception abilities to controls (Martins et al., 2019), it was not associated with gesture deficits in this study. This might be due to the nature of the stimuli we used, as neither the PKT nor the PLD task convey strong emotional states.

Some limitations should be noted for the current study. First, this is a cross-sectional study and future investigation with longitudinal studies is necessary. Second, although we corrected for current antipsychotic medication dosage, the type of antipsychotic medication, and the fact that some patients were on other medication may still influence our findings. Third, our sample includes only stable outpatients, making it difficult to generalize the current findings across all schizophrenia patients. Fourth, considering a more comprehensive visual and cognitive assessment that examines visual and motor perception impairments is important. Finally, due to the explorative nature of the study, reported correlations were uncorrected for multiple comparisons.

Taken together, the current study shows that gesture representations of static and dynamic stimuli are associated with distinct processes within the negative symptoms and motor domains in schizophrenia. Understanding the neural underpinnings involved in the processing of these two stimuli, will aid in establishing therapeutic interventions necessary to alleviate gesture deficits using noninvasive brain-stimulation (Lefebvre et al., 2020) and virtual-reality in schizophrenia patients (Pavlidou and Walther, 2021). These might be beneficial for other psychiatric disorders that exhibit similar gestural deficits to schizophrenia such as depression (Pavlidou et al., 2021; Suffel et al., 2020). Furthermore, the tasks used to test gesture perception can readily be applied in remote settings for screening/staging socio-cognitive deficits in schizophrenia.

## Funding

This study was funded by the 10.13039/501100001711Swiss National Science Foundation (#184717) given to SW.

## CRediT authorship contribution statement

AP - Conceptualization, data analyses, interpretation of results, drafted manuscript, edited manuscript. VC - Recruitment, data acquisition. LM - Clinical Assessments. SVK - Data Acquisition. SW - Conceptualization, protocol write-up, interpretation of results, edited manuscript, funding, supervision. All authors revised and approved the final version of the manuscript.

## Declaration of competing interest

SW received honoraria from Janssen, Lundbeck, Mepha, Neurolite, and Sunovion. All other authors confirm that they have no conflict of interest.
